# Gallbladder perforation associated with carcinoma of the duodenal papilla: a case report

**DOI:** 10.1186/1477-7819-8-41

**Published:** 2010-05-20

**Authors:** Akihiro Hosaka, Mikiko Nagayoshi, Katsuyoshi Sugizaki, Yukiyoshi Masaki

**Affiliations:** 1 Department of Surgery, Ome Municipal General Hospital, 16-5, Higashi Ome 4-chome, Ome-shi, Tokyo, 198-0042, Japan

## Abstract

**Background:**

Gallbladder perforation is a rare clinical condition, which mostly occurs following acute cholecystitis associated with cholelithiasis. A tumor of the ampulla of Vater causes gradually progressive symptoms, and is rarely associated with perforation of the gallbladder.

**Case Presentation:**

A 56-year-old man with carcinoma of the ampulla of Vater presented with spontaneous gallbladder perforation and localized bile peritonitis. He complained of right upper abdominal pain, and laparotomy revealed perforation of the gallbladder with no gallstones. Postoperative upper gastrointestinal endoscopy demonstrated a slightly enlarged duodenal papilla, and biopsy revealed adenocarcinoma of the ampulla. Pylorus-preserving pancreaticoduodenectomy was performed subsequently.

**Conclusion:**

Ampullary carcinoma can be associated with gallbladder perforation and present with acute manifestations. Immediate surgical treatment is required for this condition.

## Background

Gallbladder perforation (GBP) is a rare but life-threatening condition, which usually requires immediate surgical intervention. Most cases are complicated by acute cholecystitis associated with cholelithiasis [1], although acute acalculous cholecystitis or intramural vessel thrombosis can sometimes lead to GBP [2,3].

A tumor of the ampulla of Vater causes gradually progressive symptoms such as jaundice or weight loss, and rarely presents with acute manifestations [4-6]. In this report, we describe a case of ampullary carcinoma presenting with acute development of GBP and bile peritonitis, and discuss the clinical features of the disease.

## Case Presentation

**Figure 1:**
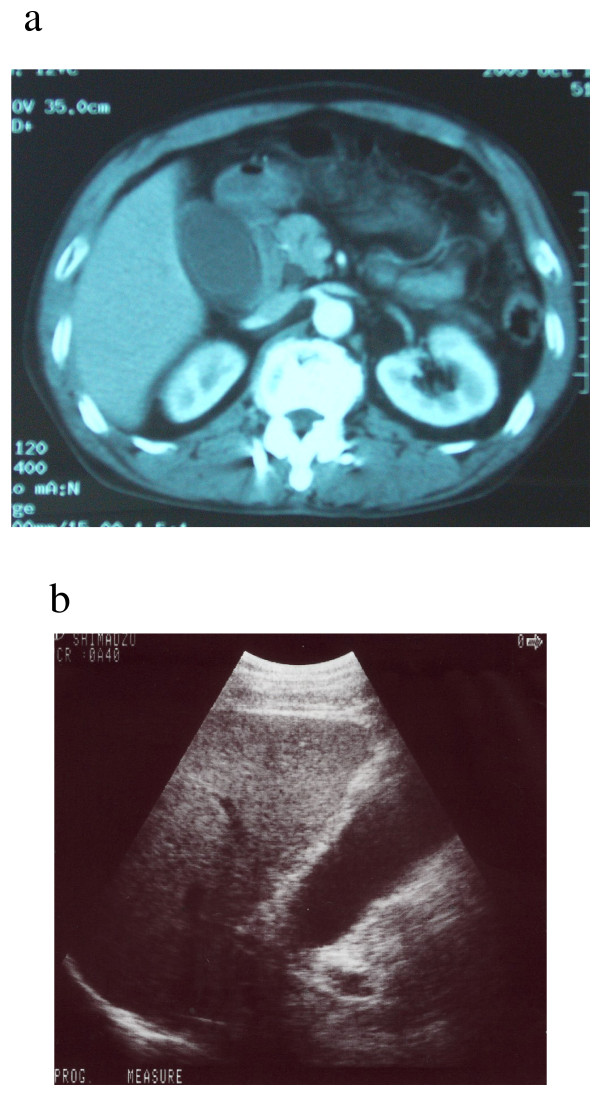
**Preoperative imaging findings.** Preoperative computed tomography (a) and echography (b) show distention of the gallbladder and thickening of its wall.

A 56-year-old man was referred to our hospital with right upper abdominal pain, which had worsened over the previous two days. He had been free of symptoms previously. He had a history of moderate smoking and alcohol consumption, and no appreciable medical or family history. On admission, his body temperature was 37.4°C. Blood examination showed a white blood cell count of 8900/mm^3^, C-reactive protein level of 0.08 mg/dl, total bilirubin level of 0.6 mg/dl, aspartate aminotransferase level of 57 IU/l, and alanine aminotransferase level of 67 IU/l. Computed tomography (CT) and echography demonstrated distention of the gallbladder and thickening of its wall and dilatation of the common bile duct, but no gallstones were detected (Fig 1).

**Figure 2:**
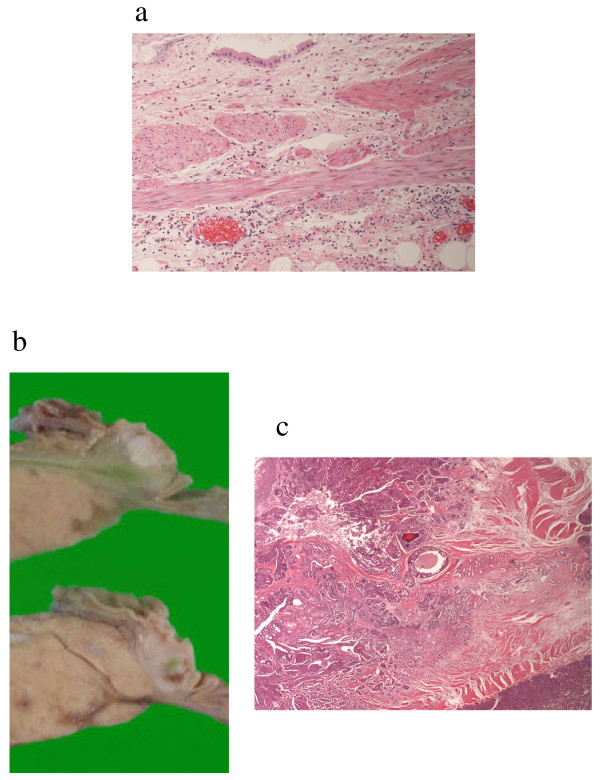
**Pathological findings.** Pathological examination of the gallbladder revealed inflammatory change of its wall (a). The tumor of the papilla was well-differentiated tubular adenocarcinoma with a maximum diameter of 1.3 cm (b, c).

He was diagnosed with acute cholecystitis, and initially treated with fluid resuscitation and administration of antibiotics. However, the abdominal pain did not improve, and laparotomy was performed the day after admission, which revealed biliary ascites around the gallbladder and partial necrotic change in the neck and body of the gallbladder. We performed cholecystectomy and intraoperative cholangiography, which revealed no stones in the gallbladder and bile duct. The postoperative course was uneventful. Pathological examination of the resected gallbladder revealed inflammatory change of its wall, and no arterial occlusive change (Fig 2a). Microbiological test of the bile showed negative results. Upper gastrointestinal endoscopy performed postoperatively showed slight enlargement of the duodenal papilla. Adenocarcinoma of the papilla was diagnosed by biopsy, and pylorus-preserving pancreaticoduodenectomy (PPPD) was performed 6 weeks after the first surgery. Intraoperative findings revealed slight dilatation of the common bile duct, and a soft pancreas with normal pancreatic duct. Pathological examination of the resected specimens demonstrated well-differentiated tubular adenocarcinoma with a maximum diameter of 1.3 cm localized in the papilla, with no lymph node metastasis, classifying the tumor as TNM stage IA (T1N0M0) (Fig 2b,c). Minor pancreatic leakage occurred during the postoperative course, which was treated conservatively. The patient has been free of recurrence during the 4-year follow-up after surgery.

## Discussion

It has been reported that GBP occurs in about 5% of patients with acute cholecystitis [1,7]. Ischemic changes of the gallbladder wall triggered by progression of local inflammation lead to gangrene and perforation, which might explain why perforation occurs in the fundus, the most distant part from the main feeding artery, in more than half of cases. Systemic vascular disorders, such as atherosclerotic cardiovascular disease and diabetes, immunosuppressed states, and malignancy are major risk factors for GBP [1]. Most cases of GBP follow an exacerbation of acute cholecystitis with cholelithiasis, and GBP without gallstones is rare. Such cases are mainly attributed to impairment of the blood supply induced by intramural thrombosis [3]. In our patient, who had no underlying risk factor for GBP, no stones were found in the gallbladder, and postoperative pathological examination revealed no thrombotic occlusion of intramural vessels, which is extremely uncommon. The cause of GBP remains unclear. It might have been induced by acute progression of cholecystitis, although the causal relationship between the ampullary tumor and GBP is not obvious.

Preoperative diagnosis of GBP is often difficult, which delays surgical intervention and leads to high morbidity and mortality [1,7]. CT and ultrasonography are useful in making the diagnosis. Gallbladder wall thickening, pericholecystic fluid collection, and a streaky omentum or mesentery are common findings of GBP [1,7-9]. However, accurate diagnosis of a defect in the gallbladder wall is rather challenging. CT has been reported to be more sensitive than ultrasonography in detecting a perforation site in the gallbladder [1,8], while Sood et al. [9] reported that a defect in the gallbladder wall could be visualized in more than 70% of cases either by CT or ultrasonography, and suggested the latter as the first-line imaging modality in the evaluation of suspected GBP cases. In our patient, the defect in the gallbladder wall and the amount of leaked bile were small, which made preoperative diagnosis difficult.

Obstructive jaundice is the most common presentation of carcinoma of the papilla, followed by weight loss, abdominal pain, and nausea [4-6]. The disease usually shows gradual progression of these symptoms, and rarely displays an acute clinical onset. Associated GBP, as in our patient, is extremely uncommon. The prognosis of ampullary carcinoma is relatively better than that of other biliary tract cancers after surgical resection [10]. Lymph node metastasis and pancreatic invasion are important prognostic factors [4,11]. Beger et al. [12] reported that lymph node involvement was observed in about 10% of patients with a pT1 carcinoma of the papilla. Therefore, Kausch-Whipple procedure or PPPD with lymph node dissection is the first choice of treatment even in patients with localized cancer, although local resection might be beneficial in patients with a poor general condition [12,13].

## Conclusion

Although extremely rare, carcinoma of the duodenal papilla can be associated with GBP and display acute manifestations. In a case of GBP without cholelithiasis, ampullary tumor should be considered as a possible underlying condition.

## Consent

Written informed consent was obtained from the patient for publication of this case report and accompanying images. A copy of the written consent is available for review by the Editor-in-Chief of this journal.

## Competing interests

The authors declare that they have no competing interests.

## Authors' contributions

AH participated in the treatment of the patient, collection of case details, literature search and drafted the manuscript. MN, KS, and YM participated in the treatment of the patient and data collection, and helped to revise the manuscript. All authors have read and approved the final manuscript.
